# Novel Antibodies Targeting MUC1-C Showed Anti-Metastasis and Growth-Inhibitory Effects on Human Breast Cancer Cells

**DOI:** 10.3390/ijms21093258

**Published:** 2020-05-05

**Authors:** Min Jung Kim, Jong Rip Choi, Nara Tae, Tae Min Wi, Kristine M. Kim, Dae Hee Kim, Eung Suk Lee

**Affiliations:** 1 Scripps Korea Antibody Institute, KNU Chuncheon Campus, Chuncheon, Gangwon 200-701, Korea; kmj@skai.or.kr (M.J.K.); cjr@skai.or.kr (J.R.C.); nalgoon@gmail.com (N.T.); wtm9272@naver.com (T.M.W.); 2 Department of Systems Immunology, College of Biomedical Science, Kangwon National University, Chuncheon, Gangwon 200-701, Korea; kmkim@kangwon.ac.kr

**Keywords:** MUC1-C, Mucin1-C terminal domain, Metastasis, survival, TNBC

## Abstract

Mucin1 (MUC1) is aberrantly glycosylated and overexpressed in various cancers, and it plays a crucial role in cancerogenesis. MUC1 is a type I membranous protein composed of α and β subunits. MUC1-α can be cleaved in cancers, exposing MUC1-β (MUC1-C). MUC1-C is involved with multiple cancer cellular functions, which makes it an attractive target for cancer treatment. However, its multifunctional mechanisms have not been fully elucidated and there has not been a successful therapeutic development against MUC1-C. Through a phage display process, we isolated the specific antibodies for the extracellular domain of MUC1-C. The relevant full IgG antibodies were produced successfully from mammalian cells and validated for their MUC1-C specificities through ELISA, dual FACS analysis, BLI assay, and confocal image analysis. In the comparison with reference antibody, elected antibodies showed characteristic bindings on target antigens. In the functionality assessment of high-ranking antibodies, SKM1-02, -13, and -20 antibodies highly inhibited invasion by triple-negative breast cancer (TNBC) cells and the SKM1-02 showed strong growth inhibition of cancer cells. Our results showed that these MUC1-C specific antibodies will be important tools for the understanding of MUC1 oncogenesis and are also highly effective therapeutic candidates against human breast cancers, especially TNBC cells.

## 1. Introduction

Abnormal overexpression of mucin1 (MUC1) in various cancers and the its interactions with several proteins related to cancer transformation have led to MUC1 as a potential oncological target [1,2,3]. MUC1 is a member of the mucin family and is composed of alpha and beta domains. The alpha domain, carrying highly repetitive sequences, is strongly glycosylated and is released in the process of mutation into cancer. In contrast, the beta domain is anchored to the cell membrane and faces the cytoplasm [4].

The C-terminal domain of MUC1 (MUC1-C) serves as a crucial molecule in the cancerogenic process through its interactions with several signaling receptors, especially receptor tyrosine kinases such as all four members of the human epidermal growth factor receptor (HER) family receptors, the fibroblast growth factor receptor-3 (FGFR-3), and the platelet-derived growth factor β (PDGF β) [5]. MUC1-C consists of a small extracellular domain (ECD), a transmembrane domain (TM), and a cytoplasmic domain (CD) with 58, 28, and 72 amino acids, respectively. Galectin-3 binds to the N-glycosylation site at MUC1-C and acts as a bridge for receptor tyrosine kinases (RTKs) [6]. The MUC1-C cytoplasmic domain plays a role in its intrinsic homodimerization and direct interactions with mediators of several signaling pathways. It is known that MUC1-C homodimers can be transported to the nucleus and mitochondria, resulting in diverse cellular behaviors such as inefficient transcription of p53 genes and the inhibition of apoptosis due to the direct binding of MUC1-C and p53 [7]; the inhibition of cell adhesion and migration resulting from the reduction of the E-cadherin/β-catenin complex [8,9,10,11]; and the loss of apical-basal polarity in breast cancer cells. Also, recent reports suggest that MUC1-C-dependent gene signatures are associated with poor outcomes in breast and lung cancers, reinforcing the importance of MUC1-C in cancer transformation [12]. There are various truncated forms of MUC1, and MUC1-C* is highly expressed in cancer. The release of pro-inflammatory cytokines such as interferon-gamma and tumor necrosis factor-alpha is known to activate several proteases (ADAM17, MMPs), whereby MUC1 is proteolytically cleaved into small extracellular domains, MUC1-C* [13,14]. MUC1-C* contains 45 amino acids (AAs) ECD (extracellular domains), and acts as a growth factor receptor for metastasis-associated proteins [15,16]. 

With all these complex functions and diverse interaction partners, MUC1-C is one of the key oncogenic receptors; it contributes to the loss of polarity in cancer cells, cancer stemness, epithelial-mesenchymal transition, epigenetic programming of cancer cells, and functions as an immune suppressor by induction of PD-L1 (programmed death-ligand 1) expression [17,18,19]. Therefore, the inhibition of MUC1-C function has been suggested as an effective anticancer strategy in diverse cancers [20,21]. Therapeutic strategies targeting MUC1-C are predominantly small molecules capable of penetrating into cells, for example, PMIP, a peptide that mimics the interaction domain of MUC1 with both epidermal growth factor receptor (EGFR) and beta-catenin [22]. Other candidates, peptide GO-201 and GO-202, block the oligomerization of MUC1-beta, and thereby prevent its nuclear and mitochondrial import. These two small molecules have been shown to induce growth arrest and death of human breast cancer cells [23]. However, the toxicity of PMIP and GO-201 peptides and their potential immunogenicity need to be investigated. A recent study used MUC1 ECD and part of alpha domain as an antigen to develop antibodies with specific anti-MUC1 activities. Most of the strategies employing therapeutic antibodies have targeted the variable number tandem repeat (VNTR) region and altered O-glycosylation sites of cancerous MUC1, and one of them failed in the phase II clinical study because of a lack of efficacy in breast cancer; however, the use of the AS1402 antibody as a drug candidate accelerated cancer research [24]. 

Antibodies that accurately target the extracellular domain of MUC1-C have not yet been developed clinically. So, we generated a recombinant MUC1-C ECD, which is critical for tumor progression, and a shorter antigen MUC1-C*. Phage display techniques were used to select antibodies that bind to the MUC1-C antigen and the surfaces of the MUC1-overexpressing cells. We confirmed their binding specificities via surface plasmon resonance (SPR), fluorescence-activated cell sorting (FACS), and confocal image analysis and compared them to those of the MUC1-C-targeting control antibody. We also evaluated the functionalities of these antibodies on the growth and invasion of MUC1-expressing breast cancer cell lines. Later, we examined the thermal stability and binding capacity of the selected antibodies as well. To understand MUC1 biology, which has diverse functions in tumor microenvironments [25], antibodies that recognize MUC1-C represent essential tools. In this study, we found that our antibodies specifically bind to MUC1-C and explored their therapeutic functionalities.

## 2. Results

### 2.1. Isolation of MUC1-C Specific Single-Chain Variable Fragments

**Figure 1:**
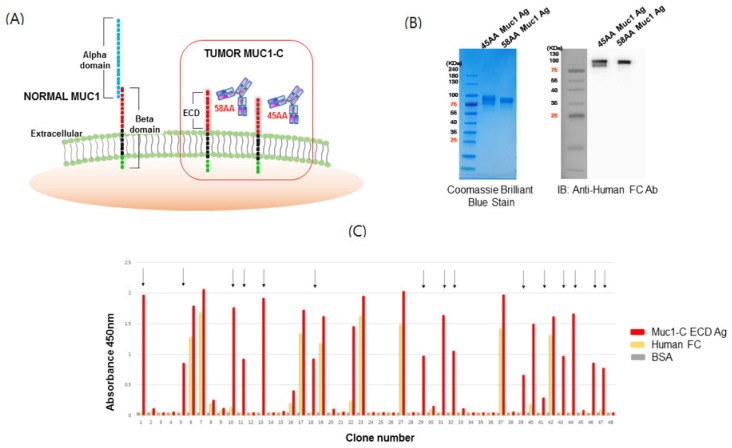
Structure of mucin1 (MUC1) and isolation of scFv clones for the MUC1-C target antigen. (**A**) Schematic structure of MUC1 and potential binding motifs on the MUC1 extracellular region. MUC1-alpha contains a signal peptide and a variable number tandem repeat (VNTR) region, which is composed of 20 amino acids that are extensively O-glycosylated at the serine and threonine residues. The beta-domain (MUC1-C) consists of an extracellular domain (ECD), transmembrane domain (TM), and cytoplasmic tail (CT) domain. The ECD of MUC1 is cleaved by metalloproteases such as ADAM17 or MMP-MT1 to generate MUC1-C (58 amino acids (AA)) or MUC1-C* (45 AA). (**B**) Purification of MUC1-C and MUC1-C* ECD target antigens. HEK293F cells were transfected with vectors encoding MUC1-C-human Fc and MUC1-C*-human Fc ECD, and after 10 days, the expressed antigens were captured from the culture media using a protein A column and stained with Coomassie brilliant blue followed by Western blot using anti-human Fc Ab. (**C**) ELISA of single-chain variable fragment (scFv) clones selected against MUC1-C ECD. A human naïve scFv antibody library precleared of Fc binders was used for biopanning with the recombinant human MUC1-C antigen. Colonies from the last panning were randomly cultured and their scFv supernatants were tested for antigen binding by ELISA. Arrows indicate scFv clones reactive to human MUC1-C ECD (58 AA) but not Fc. BSA served as a background control.

ECD full portions of MUC1-C (58 AA) and MUC1-C* (45 AA) were constructed into human Fc fusion genes by cloning into pCEP4-FC (Figure 1A). Two vectors containing each antigen were transiently transfected into 293F cells (Thermo Fisher Scientific), and then antigens were purified by Protein A column chromatography. The expression and purity of antigens was determined by SDS-PAGE and Western blotting with anti-human FC antibodies (Figure 1B). 

To screen for MUC1-C specific antibody binders, phage display was performed using the MUC1-C antigens. The human naïve antibody library was amplified and then panned using immunotubes and magnetic beads coated with MUC1-C antigens. IgG was precleared to remove non-specific binders. After four rounds of panning, periplasmic extracts derived from bacterial clones infected with individual phages were validated by ELISA against MUC1-C antigen, human Fc, and bovine serum albumin (BSA). Clones that did not express scFv were excluded through dot blot analysis using periplasmic extracts. Based on the sequence analysis of binding clones (Figure 1C), we confirmed the isolation of several independent clones via phage display panning. After the transfer of heavy chain variable domains (VH) and light chain variable domains (VL) domains of scFv into those of IgG, full-sized IgG1 were expressed and used in the following experiment. 

### 2.2. Verification of MUC1 Expression in Breast Cancer Cell Lines

**Figure 2:**
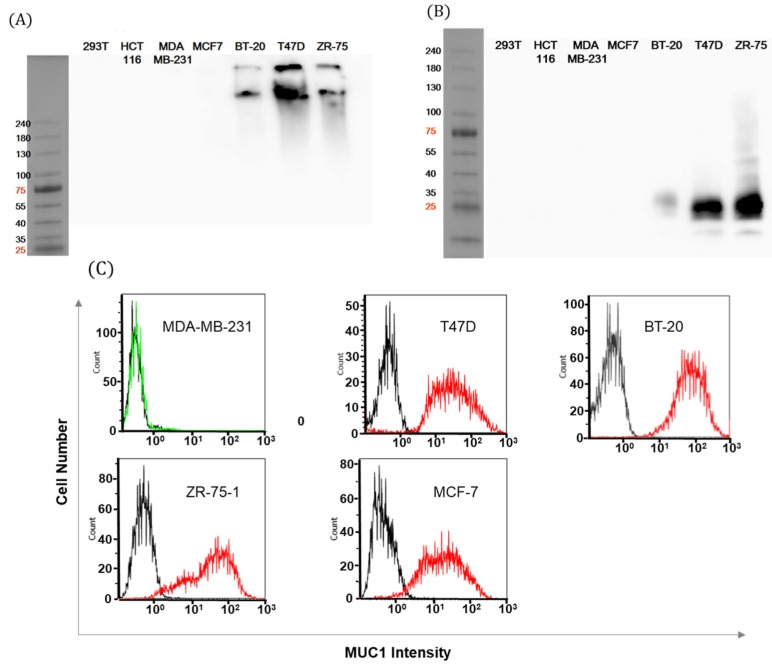
Analysis of MUC1-positive breast cancer cells. (**A**) Whole cell lysates of MUC1-positive or -negative cancer cells were separated on 4%–15% SDS-PAGE. Western blot was performed using HMPV antibody for the VNTR motif of the MUC1 alpha domain. (**B**) Cell lysates of breast cancer cells were blotted with EPR1023 Ab. The molecular weight of cleaved MUC1 was estimated at approximately 16–25 kDa. (**C**) MUC1 expression on the surfaces of breast cancer cells was measured by flow cytometry with HMPV Ab (red line) or control Ab (2nd anti-mouse Ab only, black line).

Because it is known that MUC1 expression has considerable heterogeneity not only between cell lines but also within several individual lines depending on the assays and the interacting antibody’s epitope [26], several representative cell lines were selected and their feasibility in our studies was determined [27]. MUC1 expression in various breast cancer cells showed the relevant expression patterns (Table S1). Antibodies recognizing the alpha, beta, and cytoplasmic tail of MUC1 (Table 1) were tested against the following representative breast cancer cell lines: MCF-7, BT-20, ZR-75-1, T47D, and MDA-MB-231. The expression of complete MUC1 was evaluated using HMPV Ab (Figure 2A) and the expression of MUC1-C form was confirmed using an antibody recognizing the MUC1 cytoplasmic domain (Figure 2B). Heterogeneously glycosylated MUC1-C displayed multiple bands (16–25 KDa).

Finally, the expression of the natural form of MUC1 (using the same antibody as A) in breast cancer cells was verified by FACS (Figure 2C). Other cancer cell lines also revealed MUC1 expression (Figure S1). Expression patterns of MUC1 on the cell surface were consistent with protein levels from the Western blot. We confirmed that MUC1 showed robust, high expressions in T47D, BT-20, and ZR-75-1 cell lines while MCF-7 cell showed positive FACS signals and negative immunoblot signals in our assay settings. To keep the robustness of the experiments, these cell lines with high levels of MUC1 expression were used throughout the following antibody development process.

### 2.3. SKM1 IgG Antibodies Specifically Interact with MUC1-C in Breast Cancer Cells.

**Figure 3:**
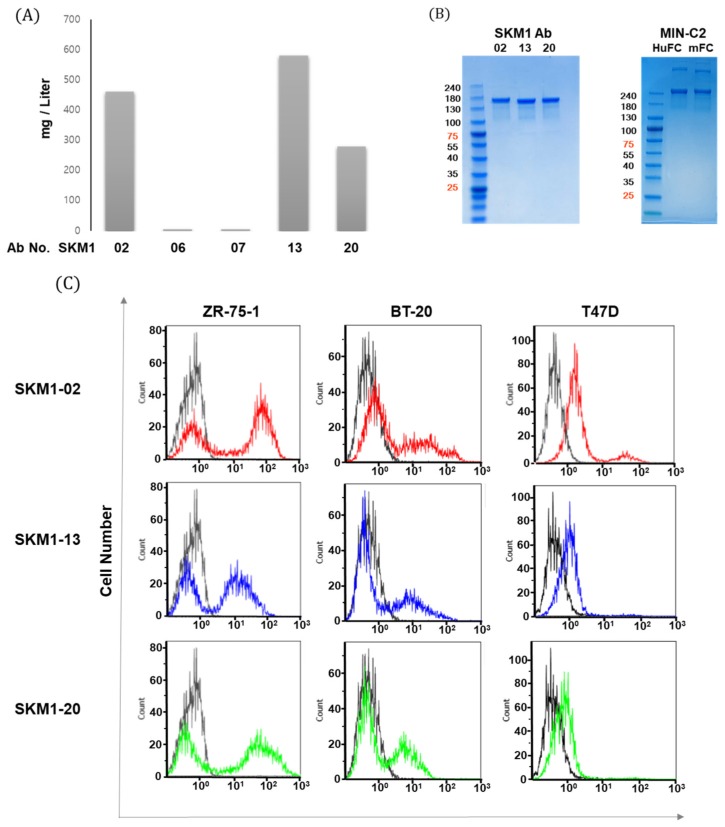
MUC1-C specific antibody generation and binding of MUC1 expressed breast cancer cells. (**A**) Comparison of transient expression levels of different IgG constructs in Expi-CHO cells harvested after 12 days, the antibodies were purified via protein A column chromatography. Each IgG antibody is expressed as mg per 1 L culture. (**B**) Coomassie blue staining showed purified SKM1 antibodies (left panel) and MIN-C2 antibody (right panel). Antibodies transiently expressed from Expi-CHO cells were purified after 12 days using a protein A column and showed expression with SDS-PAGE. For comparison with the reference antibody, MIN-C2 developed by Minerva Biotechnologies was constructed and expressed as IgG1 with either Human Fc or Mouse Fc sequences. (**C**) Specific interaction between SKM1-02, -13, or -20 Ab and MUC1 on breast cancer cells. MUC1 levels in breast cancer were measured by flow cytometry with 2nd antibody (black line) or candidate antibodies; SKM1-02 (red line), SKM1-13 (blue line), SKM1-20 (green line).

The scFv clones selected by phage display were converted to IgG1 format antibodies and produced from mammalian expression systems. Each clone was subjected to FACS analysis on MUC1 expressing cell lines such as MCF-7 and ZR-75-1 (Figure S2). Among the five clones binding strongly to cell surface, two clones (SKM1-06, 07) showed low levels of expression, distinct from the candidates. Three clones that showed robust expression, including SKM1-02, -13, and -20 (Figure 3A), were purified and analyzed with PAGE (Figure 3B) and Western blot. In order to compare the binding specificities and functionalities of the selected clones, we generated a MUC1-C*-targeted MIN-C2 antibody. A MIN-C2 antibody was engineered based on the cDNA sequence, which was generated against the membrane-proximal 45-residue peptide (from Gly^1110^ to Ala^1154^) of MUC1-C*. It specifically recognized all membrane-bound MUC1 products with different cleavage sites [15]. Two formats of control antibody were constructed in which human IgG1 heavy and kappa constant domains (HuFc) or mouse IgG2a heavy and kappa constant domains (mFc) were fused with the VH and VL domains of the MIN-C2 antibody (Figure 3B).

Next, clones SKM1-02, SKM1-13, and SKM1-20 were subjected to FACS analysis to confirm whether these antibodies can bind to the surface of ZR-75-1, BT-20, and T47D breast cancer cell lines (Figure 3C). Compared to the SKM1-13 and SKM1-20 antibodies, the SKM1-02 antibody showed the highest binding capability. The T47D cells showed single peak shifts in population, but ZR-75-1 and BT-20 cell lines showed shift patterns with distinct dual peaks. These separate peaks from ZR-75-1 and BT-20 cells appeared throughout several repeated experiments, which was similar to MIN-C2′s antigen binding in its patent. This result may be caused by diverse MUC1-C expression in each cancer cell line. Overall, these results indicate that the SKM1 antibodies specifically interact with cell surface-expressed MUC1-C in breast cancer cells, while the exact mechanism for dual peak staining has not been elucidated.

### 2.4. Specific Interaction of SKM1-Antibodies with MUC1-C and MUC1-C*

**Figure 4:**
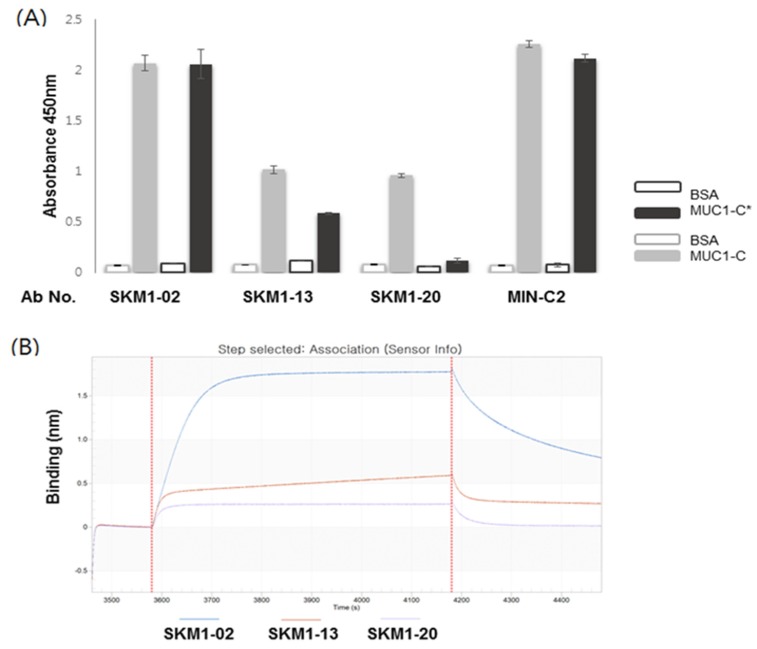
Binding of the SKM1-antibodies with MUC1-C and MUC-1*. (**A**) ELISA represented binding of hMUC1 antibodies with MUC1-C (58 AA) and MUC1-C*(45 AA). Antigens were coated onto 96-well microtiter plates, and then antibodies were incubated. Signals were detected by HRP-conjugated anti-human IgG antibody with colorimetric reagents described in the Materials and Methods. BSA was used as a background control. (**B**) Determination of antibody-binding specificities against MUC1-C antigen using ForteBio’s Octet system. The MUC1-C protein was immobilized on an AR2G sensor chip and three antibody clones showed increased sensorgrams via Octet analysis, reconfirming the characteristic binding on the MUC1-C antigen.

The binding specificity of three clones was investigated in ELISA using MUC1-C (58 AA) and MUC1-C* (45 AA) antigens. The SKM1-02 antibody strongly bound to both antigens, whereas the other two clones showed relatively low binding with both antigens, and interestingly, the SKM1-20 clone showed MUC1-C (58 AA)-specific binding (Figure 4A). The MIN-C2 antibody also displayed high immunoreactive signals against two antigens.

Finally, a direct interaction between the antibody and MUC1-C antigen was confirmed via SPR-based ForteBio’s Octet system (Figure 4B). The incubation of MUC1-C antigen coated on an AR2G sensor chip with antibody solutions generated characteristic, enhanced Octet sensorgrams, which showed MUC1-C-specific bindings of each antibody and correlation with the ELISA results. These results suggest that the SKM1 antibodies directly bound to human recombinant MUC1-C ECD antigen and SKM1-02 antibody possess superior binding capability compared with the other antibodies.

### 2.5. SKM1-02 Antibody Binds to Membrane-Proximal MUC1-C on the Cell Surface

**Figure 5:**
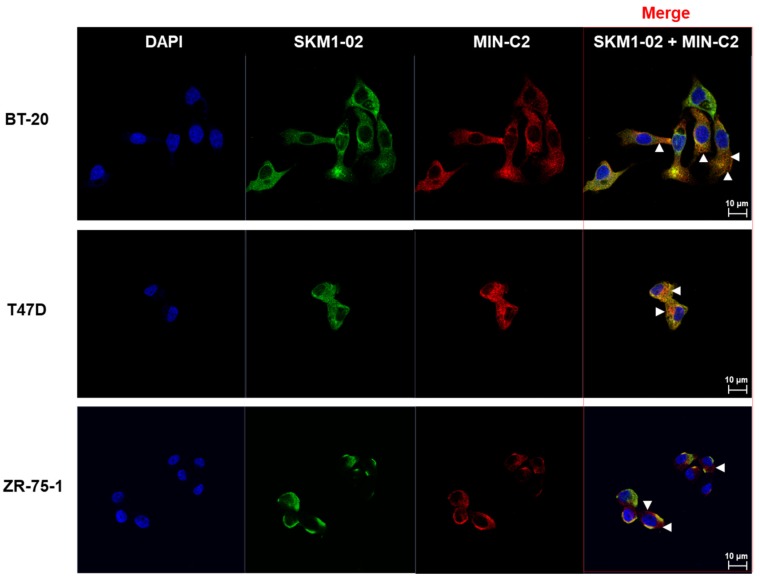
BT-20, T47D, and ZR75-1 cells were surface-stained with SKM1-02 Ab and/or MIN-C2 (mouse IgG2a version) Ab. The samples were stained using anti-human IgG Ab conjugated with Alexa 488 and anti-mouse IgG Ab conjugated with Alexa 647 secondary antibodies, respectively. Images were visualized by confocal microscopy (CLSM system; LSM 700, Carl Zeiss, Jena, Germany). Scale bar, 10 µm. FACS analysis was performed by SKM1-02 and/or MIN-C2 (MUC1-C*) with a mouse IgG constant region.

Immunofluorescence stainings of BT-20, T47D, and ZR-75-1 cell lines with SKM1-02 Ab and MIN-C2 Ab were performed to observe cell surface bindings of the antibodies (Figure 5). The antibodies and respective secondary antibodies stained the non-perforated cells. The stained regions for each antibody were merged revealing the binding on similar regions. In addition, although the degrees of staining differed, the overall staining patterns were similar between cell lines. Our results suggest that SKM1-02 and MIN-C2 antibodies share similar binding sites on the breast cancer cell surface.

### 2.6. MUC1-C Specific Antibodies Strongly Inhibit Invasive Activity of Breast Cancer Cells

**Figure 6:**
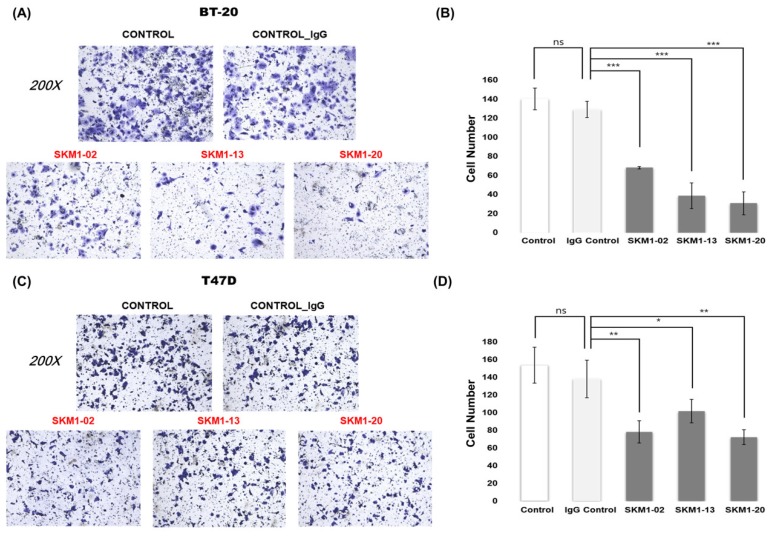
Effect of MUC1-C targeting antibodies on metastatic activity. To analyze the anti-metastatic function of selected antibodies, in vitro Matrigel invasion assays of BT-20 and T47D cells were performed. (**A**,**C**) Representative images for Matrigel invasion assay for the antibody treatments on BT-20 and T47D cells. After BT-20 and T47D cells were incubated for 48 h with 10 µg/mL anti-MUC1-C antibodies and IgG control, the cells migrated to the lower chamber were fixed, stained with crystal violet, and counted using inverted microscopy(magnification 200×). Random fields were scanned (three fields per filter of the well) for accurate measurements. (**B**,**D**) The average number of total cells that migrated to the lower chamber per field of view in three independent experiments for BT-20 and T47D cells were plotted. ns: not significant (*p* > 0.05), * *p* ≤ 0.05, ** *p* ≤ 0.01, *** *p* ≤ 0.001.

The MUC1-C domain is thought to be a key regulator of cancer invasion [28,29,30]. An invasion chamber was used to test whether SKM1 antibodies affected the metastatic invasion of cancer cells (Figure 6). In the case of the non-invasive cell line, ZR-75-1, few cells passed through the chamber. However, in our invasion assay setting for 24 hr of antibody incubation for T47D and BT20 cells, all the SKM1 antibodies (-2, -13, and -20, treated as 10 µg/mL) showed fairly effective invasion inhibitions compared to the control groups. BT-20 cells showed more sensitive inhibitions (Figure 6A,B) than T47D cells (Figure 6C,D). Regarding the possibility of invasion interference from the changes of cell growth rate, it was unlikely for the tested cell lines to show such levels of invasion inhibition within 24 hr, which have relatively long doubling time (BT20: 66~70 hr, T47D: 32~56 hr, ZR-75-1: 54~80 hr from Expasy Cellosaurus web Database). This result suggests that the SKM1 antibodies play critical inhibitory roles in the invasion of MUC1-expressing breast cancer cells.

### 2.7. SKM1-02 Antibody Reduces the Viability of Breast Cancer Cells

**Figure 7:**
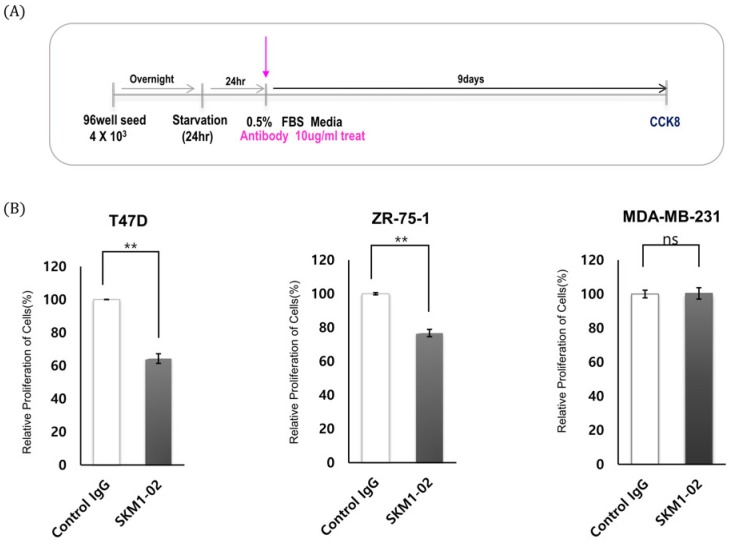
Effect of SKM1-02 antibody on proliferation of breast cancer cells. (**A**,**B**) T47D and ZR-75-1 cells (MUC1-positive) and MDA-MB-231 cells (MUC1-negative) were treated with the anti-hMUC1 monoclonal antibody 10 µg/mL or control IgG. Cell proliferation was evaluated using a CCK-8 assay on day 9 post-treatment. The data are based on 3 independent trials; ns: not significant (*p* > 0.05). ** *p* ≤ 0.01.

MUC1-C expression induces cell growth and tumorigenicity, and, therefore, the effect of MUC1-C-binding antibody on cancer cells was tested. As shown in Figure 7, proliferation assays of breast cancer cells were designed, and the cell viability was measured using a CCK-8 assay. Treatment with 1 µg/mL of antibody showed minimal effect, whereas 10 µg/mL of antibody showed significantly inhibited growth rates of breast cancer cells: T47D cells (~28%) and ZR-75-1 cells (~ 25%). As expected, SKM1-02 antibody did not affect the cell growth of the MUC1-negative MDA-MB-231 cell line. These results suggest that the MUC1-C-targeting SKM1-02 antibody inhibited the cell viability of MUC1-expressing breast cancer cells.

### 2.8. Thermal Stability and Binding Affinity of SKM1-02 Antibody

**Figure 8:**
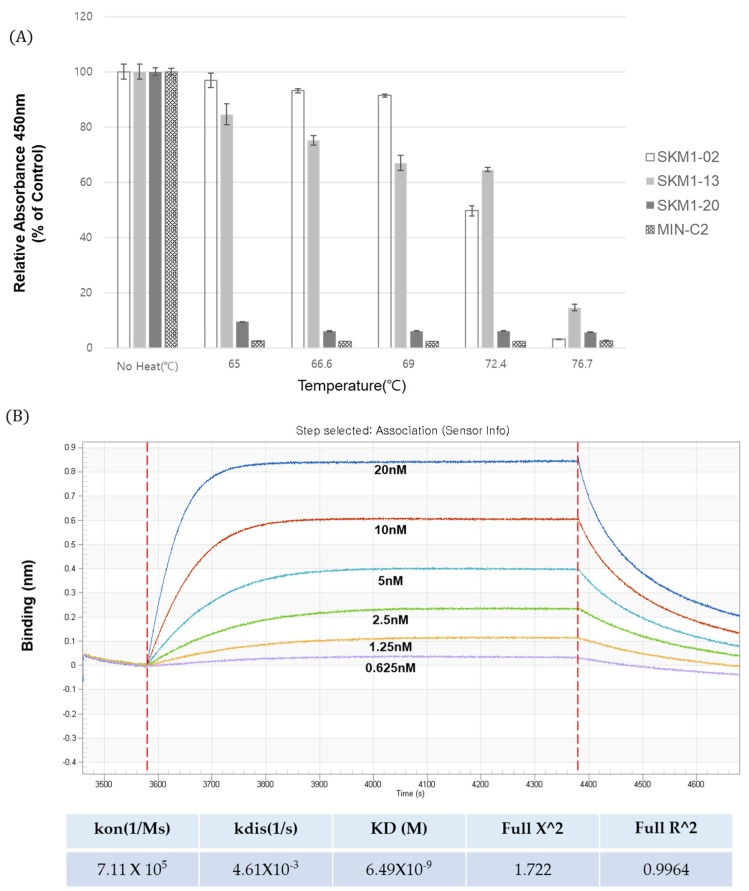
Thermal stability and binding affinity of anti-MUC1 antibodies. (**A**) Thermal stability testing of candidate antibodies. Anti-MUC1 antibody samples were incubated at incremental temperatures (65 °C~76.7 °C) for 10 min in a gradient PCR machine, and tested for binding to the MUC1 antigen in an ELISA assay. (**B**) The binding affinity of SKM1-02 with MUC1-C Ag (58AA ECD) was measured via biolayer interferometry using the Octet^®^ RED96 system. Increasing amounts of MUC1-C antigen were immobilized on an AR2G sensor chip, and antibodies were added. KD = equilibrium dissociation constant; K_on_ = association rate constant; and K_off_ = dissociation rate constant.

To explore the feasibility of the SKM1-02 antibody as a therapeutic drug, its thermal stability and affinity were tested. The thermal stability of SKM1 samples was tested at five temperatures ranging from 65 °C to 76.7 °C and each heated sample was analyzed with ELISA against the MUC1-C antigen (Figure 8A). The results showed stable binding of the SKM1-02 antibody up to 72.4 °C and a sharp decline in binding after 76.7 °C. The stability of the SKM1-13 antibody was comparable to that of other MUC1-C binders. SKM1-20 and MIN-C2 showed low binding even after incubation at 65 °C. We found that the SKM1-02 antibody has a highly stable structure. 

The affinity of the SKM1-02 antibody was assayed using biolayer interferometry (BLI) with an Octet^®^ RED96 system (Figure 8B). Following the immobilization of the MUC1-C antigen on the AR2G sensor chip (5 µg/mL), serially diluted SKM1-02 samples were applied to the Octet instrument. The binding curves increased in a concentration-dependent manner, with a dissociation constant (Kd) of 6.49 nM.

Based on the expression, thermal stability, binding affinity, and novel inhibitory function in cancer cell proliferation and invasion, the SKM1-02 antibody showed MUC1-C-specific binding, novel function, and potential as a therapeutic candidate.

## 3. Discussion

We generated antigens mimicking the ECD of MUC1-C (58 AA) and MUC1-C* (45 AA) as human Fc-fused forms to leverage their natural conformation in antibody screening, and antibody phage panning was performed to isolate MUC1-C specific antibodies. Phage display antibody panning resulted in the isolation of 24 human antibodies with binding specificities against the MUC1-C antigen. To confirm their specificities and functionalities, they were produced as full-sized IgG1 antibodies and used in several immunological assays. For functional reference, the MIN-C2 antibody with specific binding to MUC1-C was used in every assay.

The top three clones including SKM1-02, -13, and -20 with a relatively high level of expression in a mammalian cell system reconfirmed MUC1-C-specific binding as full-size IgG in several immunological assays, such as ELISA, FACS assay, and BLI using the Octet system. Interestingly, SKM1-02 Ab showed similar binding with the 58 AA ECD and 45 AA ECD of MUC1-C; however, the -13 and -20 clones showed slightly different bindings with two antigens; the -20 clone showed an almost exclusive binding to the 58 AA ECD in the ELISA. Octet analysis also showed the same results as the ELISA. Confocal image analysis also showed the overlapped staining of SKM1-02 and MIN-C2 antibodies, which suggested that our MUC1-C-targeting antibodies bind to the MUC1 beta ECD domain and each antibody has slightly different epitopes within the MUC1-C ECD domains. The effects on MUC1-C function due to differences of the antibodies’ epitopes are not clear yet. However, since the results of the migration and growth inhibition assay showed certain differences between the antibodies, it is necessary to study more detailed mechanisms between these antibodies and MUC1-C.

In addition, the MUC1-C binding antibodies to some cancer cells resulted in two separate peaks. The T47D cell line showed no peak separation but ZR-75-1 and BT-20 cell lines showed dual peaks. This dual peak staining pattern was also shown from the FACS staining of the reference antibody, MIN-C2. We tried the characterization of individual populations through cellular sorting by a BD FACSAria instrument, which revealed that the two distinct peaks reappeared from each population (data not shown), which suggested that these peaks represent two groups of cells engaged in cell proliferation. Further, the two distinct peaks may represent a mixture of cancer stem cells and normal cancer cells; however, no match was found with stem cell markers. Further experiments are needed to understand the high and low peaks in the MUC1-C-specific antibody staining. 

No antibody targeting of MUC1 has been successful in clinical studies so far, most of which targeted the alpha domain of MUC1, because cancer-specific underglycosylated MUC1 plays a significant role in carcinogenesis and most of the antibodies obtained using hybridoma approaches against MUC1 generated alpha-domain-targeting antibodies. Since the first pioneered candidate AS1402 failed in a phase II clinical study because of its cancer-promoting effect [24], alternative approaches such as drug-conjugates, peptides, or vaccines have been investigated, but few have used MUC1-C*-targeting monoclonal antibodies. 

To address the potential of therapeutic antibody development targeting the MUC1-C extracellular domain, we tested the functionality of the top three clones in assays for cancer cell invasion and cancer growth inhibition. All three antibodies showed high levels of migration inhibition, which varied in cell lines and individual antibodies. It is known that MUC1-C translocation to the nucleus in association with beta-catenin represses E-cadherin expression, which then destabilizes the adherens junctions and induces cytoskeletal rearrangement resulting in loss of cell–cell contact inhibition. Therefore, MUC1-C antibody treatment affects all these events and leads to anti-metastatic outcomes [31]. The results of the invasion assay against the BT-20 cell line (TNBC, triple-negative breast cancer) and T47D cell line (her2 negative cell line) showed that these antibodies represent potential anti-metastatic therapeutic candidates. In addition, the proliferation assay for SKM1-02 showed promising results. Because MUC1-C is involved in multiple mechanisms of cell proliferation, such as extracellular bridging through galectin 3 and RTKs, homodimer formation and interaction with signal mediators at cytoplasmic domain, and activation of transcription factors during the nuclear and mitochondrial translocation of MUC1-C, the results of the SKM1-02 proliferation inhibition assay cannot be explained currently; however, based on one of these mechanisms, it exerts growth inhibition. From a drug development perspective, with a dissociation constant of 6.5 nM against MUC1-C and a binding capability even at 72.4 °C, SKM1-02 represents a potential antibody drug candidate.

Currently, several antibodies against cancer-specific antigens are being tested on the CAR-T cell platform, which showed that the epitope of the antibody is one of the key factors contributing to robust anticancer activities. The MUC1 antigen has been analyzed comprehensively in CAR-T therapy, but the antibody used in CAR-T cell targeting yielded different efficacies. Therefore, MUC1-C-targeting antibodies such as SKM1-02 that contain membrane-proximal epitopes for CAR-T cells represent the optimum antibody candidate for the development of CAR-T therapy. 

## 4. Materials and Methods

### 4.1. Phage Display

Phage display experiments were mainly performed with the protocols that are well-described in detail in the “Phage Display: A Laboratory Manual” [32]. Briefly, biopanning for the enrichment of antibody binders was performed using immunotubes (maxisorp, Nunc, Rochester, NY, USA) or magnetic beads (Dynabeads M-270 epoxy, Invitrogen, Invitrogen, Carlsbad, CA, USA) that were either coated with human normal IgG or recombinant MUC1-C protein. Phages were pre-adsorbed on human IgG coated beads to remove human Fc binders, then the phages in the supernatant were incubated at 4 °C for 1 h with immobilized antigens. The phage-antigens were washed 5 times with 0.1% Tween 20/PBS and the bound phages were eluted with 0.25% trypsin (Gibco, Waltham, MA, USA). The eluted phages were amplified by direct infection to *E. coli* ER2738 in the exponential growth phase. The phages from an overnight culture were purified by PEG precipitation (final 5% *w*/*v* polyethylene glycol 8000 (PEG8000), 0.5 M NaCl) and used in the next panning cycle. Four rounds of panning were performed to enrich the antibody binders. Individual colonies from the 3rd and 4th rounds of panning were picked randomly and tested by enzyme-linked immunosorbent assay (ELISA) for MUC1-C antigen binding and the clones with positive ELISA signal were applied for DNA sequencing.

### 4.2. ScFv ELISA

A total of 600 phage clones were tested for reactivity with MUC1-C and MUC1-C* by ELISA. Single colonies from 3rd and 4th panning output plates were cultured in a 96-deep-well plate (300 μL media/well with ampicillin) with 1M IPTG induction and shaking at 25 °C overnight. The next day, bacterial cells were spun down, and the supernatant was removed. The pellet was suspended with 80 μL ice-cold 1X TES buffer (20% *w*/*v* sucrose, 50 mM Tris, 1 mM EDTA, pH 8.0) and incubated on ice for 30 min. Then, 120 μL of ice-cold 0.2X TES buffer was added to each well, mixed thoroughly, and incubated on ice for 30 min. After spinning down, the supernatant contained periplasmic fraction. Periplasmic extracts containing scFv were added to ELISA plates coated with 2 µg/mL antigen or control human normal IgG in phosphate-buffered saline (PBS). The plate was incubated at room temperature for 1 h and washed 5 times with 0.05% Tween/PBS, followed by incubation with anti-Myc antibody (3E10 clone) in 5% skim milk/PBS for 1 hr. After washing and incubation with anti-mouse IgG-HRP secondary antibody for 1hr, the well with antibody binding was visualized with a general TMB substrate colorization method.

### 4.3. Immunoblotting

Cells were lysed in RIPA buffer (50 mM Tris-HCl, 150 mM NaCl, 1% NP-40, and 0.25% Na-deoxycholate) with a protease and phosphatase inhibitor cocktail (Genedepot, Katy, TX, USA). The concentration of total protein was determined using the BCA Assay kit (Thermo Scientific, Rockford, IL, USA). Whole-cell lysates were separated by SDS-PAGE and transferred to PVDF membranes (Bio-Rad, Hercules, CA, USA). The primary antibodies used were anti-MUC1 (BD Bioscience, Franklin Lakes, NJ, USA) and anti-MUC1-C (AbCam, Cambridge, UK). Antibodies were routinely used at 1∶1000 dilution with 5% skim milk/PBS for primary antigen binding, then secondary anti-mouse IgG conjugated with horseradish peroxidase were incubated at 1:5000 dilution with 5% skim milk/PBS. This immunoblot was visualized with a chemiluminescent Western blot detection kit (Ab Frontier, Seoul, Korea) and Luminescent Image Analyzer (GE Healthcare Bio-sciences, Chicago, IL, USA) according to the manufacturer’s instructions.

### 4.4. SPR (OCTET)

Binding affinity of the anti-hMUC1 monoclonal antibody was determined using an Octet RED96 system (ForteBio, Pall Life Sciences, Westborough, MA, USA) and the experiment was performed in accordance to manufacturer’s protocol. All samples were diluted in running buffer. Wells of a black flat bottom polypropylene plate were loaded with each sample and the reaction buffers. Recombinant hMUC1-antigen was immobilized onto amine-reactive biosensors (AR2G, ForteBio, Fremont, CA, USA). The MUC1-C antibody was serially diluted two-fold (0.626–20 nM) in a kinetic buffer. Kinetic parameters and dissociation constant of the antibody were calculated with data analysis software (ver9) provided by the Octet system of ForteBio, in which antigen/antibody binding was modeled as 1:2 binding.

### 4.5. Cell Culture

Cancer cell lines were maintained at 37 °C with 5% CO_2_. ZR-75-1 was obtained from American Type Culture Collection (Manassas, VA, USA) and T47D, BT-20, MCF-7, HCT116, and MDA-MB-231 were obtained from KCLB (Korean Cell Bank, Seoul, Korea). ZR-75-1 and T47D cell lines were cultured in RPMI 1640 (Hyclone, Logan, UT, USA) medium supplemented with 100 units/mL penicillin, 100 μg/mL streptomycin, and 2 mmol/L l-glutamine. All the above cell culture reagents were purchased from Invitrogen (Carlsbad, CA, USA). BT-20 (Korean Cell Bank, Seoul, Korea) cells were cultured in Eagle’s minimum essential medium (Gibco, Gaithersburg, MD, USA) supplemented with 10% (*v*/*v*) fetal bovine serum (FBS; Gibco) as guided by ATCC. HEK293F cells were cultured in Freestyle^TM^ expression medium (Gibco) using Multitron incubation shaker (Infors HT, Bottmingen, Switzerland) with 8% CO2, 37 °C.

### 4.6. Flow Cytometry (FACS)

Flow cytometry was performed in a FACS calibur (Becton-Dickinson, BD Biosciences) equipped with a 488 nm argon laser and a 615 nm red diode laser. Cells were harvested and cleaned with FACS buffer (2% FBS in PBS). The cells were incubated with the recommended IgG (4 µg/mL) for 1 h at room temperature. After washing the unbound antibody, followed by incubation with Alexa Fluor 488-conjugated goat anti-Human IgG antibody or 647 conjugated goat anti-mouse IgG antibody for 1 h, the samples were analyzed using Kaluza software (Beckman Coulter, Brea, CA, USA).

### 4.7. ELISA

A 50 µl aliquot of antigen (2 µg/mL) in PBS buffer was added to the individual wells of a microtiter plate and incubated overnight at 4 °C. The coating solution was removed and the plate was washed three times by filling the wells with 100 μL PBS containing 0.05% Tween 20 (PBS-T). The remaining protein-binding sites in the coated wells were blocked by adding 100 μL of the blocking buffer, 3% BSA in PBS to each well, and incubated for 1 h at RT with gentle shaking. The plates were then incubated with 1 µg of IgG or anti-MUC1-C clones in blocking buffer for 1 h at 37 °C, then were washed three times with PBS-T, followed by secondary antibody incubation with (HRP)-conjugated goat anti-human IgG antibody for 1 h. The bound antibody was detected by the addition of the 3,3′,5,5′-tetramethylbenzidine substrate solution (TMB; BD Biosciences, San Jose, CA, USA). After incubation for 5 min, the color development was stopped with 1N HCl. The absorbance (optical density at 450 nm) of each well was determined with a plate reader.

### 4.8. Proliferation Assay

A total of approximately 4 × 10^3^ cells were plated in 96-well plates in triplicate and cultured in 90 μL medium containing 10% FBS. After 1, 3, 6, and 9 days of incubation with monoclonal antibody, a 10 μL aliquot of cell proliferation assay reagent from Cell Counting Kit-8 (Dojindo, Kumamoto, Japan) was added to each well and they were left for 1–4 hrs of additional incubation in a CO_2_ incubator. Final absorbance was measured at 450 nm using the microplate reader Epoch (Thermo Fisher, Waltham, MA, USA). Each experiment was repeated three times.

### 4.9. Purification of Recombinant Human MUC1-C Protein and Full IgG Antibodies

The human Muc1 antigen containing 45 and 58 amino acids (45 AA: Glycine from 1110 to alanine 1154, 58 AA: serine from 1097 to alanine 1154; GenBank Accession No. P15941) was synthesized and amplified by PCR using the following primer sets: 58 AA sense 5ʹ-AGGCCCAGGCGGCCTCTGTGGTGGTACAA-3′ and anti-sense 5ʹ-AGGCCAGCCAGGCCCCCAGCCCCAGACTG-3′, 45 AA sense 5ʹ-GGGGCCC AGGCGGCCGGTACCATCAATGTCCACGAC-3′ and anti-sense 5ʹ- GCTGGCCAGCCAGGCCAGC CCCAGACTGGGCAGAG-3′. Using the Sfi I restriction enzyme, the amplified DNA fragments were cloned into the expression vector pCEP4-FC. The MUC1-pCEP4 vector was transiently expressed in 293F cells and after 12 days, the culture medium was harvested.

Each set of VH and VL primers for the 24 MUC1-C binding clones were synthesized and used for PCR-amplification of individual VH and VL fragments (Table S2). Each VH primer was designed to have restriction sites for KpnI or BamHI at 5′ ends and NheI at 3′ ends. For VL primers, BamHI and BsiWI restriction sites were incorporated at the 5′ and 3′ ends, respectively. VH and VL fragments of each clone were subcloned into the pCEP4 mammalian expression vector (Invitrogen) as a fused form either with CH1-CH2-CH3 for heavy chain expression or with C_kappa_ for light chain expression. Matching heavy and light chain vectors for each clone were co-transfected into Expi-CHO cells according to manufacturer’s protocol and after 12 days, the culture medium was harvested. The MUC1 antigens and full IgG antibodies were purified by affinity column chromatography using protein A agarose beads (GenScript, Piscataway, NJ, USA).

After dialysis with phosphate-buffered saline at pH 7.4 (PBS), antibody concentration was quantified using a NanoDrop 2000 spectrophotometer (Thermo Fisher Scientific, Waltham, MA, USA), and the purity of each antibody was evaluated by SDS/polyacrylamide gel electrophoresis (PAGE) and Coomassie brilliant blue staining.

### 4.10. Immunocytochemistry and Confocal Microscopy

The cells were seeded on sterile glass coverslips in 12-well culture plates and incubated in freshly prepared 4% paraformaldehyde-PBS at room temperature for 10 min. The MUC1-C antibody dilutions were prepared in 1% normal BSA. The coverslips were incubated for 1 h at room temperature. After rinsing the unbound monoclonal antibody, the slides were incubated with Alexa Fluor-conjugated secondary antibodies, such as Alexa Fluor 488-conjugated goat anti-Human IgG antibody or 647 conjugated goat anti-mouse IgG antibody, for 1 h. All the samples were mounted and observed using confocal laser scanning microscopy (CLSM system; LSM 700, Carl Zeiss, Jena, Germany).

### 4.11. Invasion Assay

BD Matrigel Basement Membrane Matrix (BD BioScience, concentration approx. 10 mg/mL) was mixed 1∶4 with PBS and allowed to polymerize in transwell inserts (Corning, Corning, NY, USA) overnight and 3×10^5^ cells were seeded directly onto the Matrigel-coated inserts. Transwell inserts were finally placed in serum-free medium, and the lower compartment was filled with a medium supplemented with 10% FBS (to obtain a chemotactic gradient). Human MUC1-C candidate Abs were added to the upper compartment of the invasion chamber, and then incubated overnight. At least three independent experiments were performed in duplicate for each sample. After incubation for 48 h, the chambers were washed with phosphate-buffered saline (PBS) and fixed with 4% paraformaldehyde in PBS. Samples were then washed and stained with crystal violet. The cells passing through the filter into the lower chamber were counted using a phase-contrast microscope.

## 5. Conclusions

Phage display panning using the MUC1-C extracellular domain resulted in successful isolation of several MUC1-C-specific antibodies that can be used as important tools to elucidate complex and dominant mechanisms of MUC1 oncogenesis and establish their therapeutic potential based on anti-metastatic effect and cancer proliferation inhibition.

## Figures and Tables

**Table 1:** Antibody recognition for MUC1 of each domain.

Antibody	Recognize Sites	MW
HMPV	Anti-MUC1 α domain (VNTR motif)	<150 KDa
MIN-C2	Anti-MUC1 β domain (MUC1-C*, 45 AA ECD)	-
EPR1023	Anti-MUC1 Cytoplasmic Tail (CT)	17~25 KDa

MUC1 protein is composed of α (variable number of tandem repeats, VNTR) and β domains, and a cytoplasmic tail (CT). HMPV, MIN-C2, and EPR1023 recognize α, β, and CT domains of MUC1, respectively.

## Supplementary Materials

Supplementary materials can be found at https://www.mdpi.com/1422-0067/21/9/3258/s1.

## Abbreviations

MUC1	mucin1
mAb	monoclonal antibody
HER2	human epithelial receptor 2
ECD	extracellular domain
CD	cytoplasmic domain
scFV	single-chain variable fragment
VH	heavy chain variable domains
VL	light chain variable domains
CH1/2/3	heavy chain constant domains 1/2/3
Fc	fragment crystallizable region
IgG	immunoglobulin G
TNBC	triple-negative breast cancer
FGFR-3	fibroblast growth factor receptor-3
PDGF	platelet-derived growth factor β
RTK	receptor tyrosine kinase
ADAM17	ADAM metallopeptidase domain 17
MMP	matrix metallopeptidases
AA	amino acids
