# Supplementary material for: Novel Antibodies Targeting MUC1-C Showed Anti-Metastasis and Growth-Inhibitory Effects on Human Breast Cancer Cells
Source: Int J Mol Sci. 2020 May 5;21(9):3258. doi: 10.3390/ijms21093258 (PMC7247325; doi:10.3390/ijms21093258)
Supplement: Supplementary file 1 [file ijms-21-03258-s001.pdf]

Table S1. Classification of Breast Cancer Cell Lines based on Molecular Subtype.

Cell Lines	ER	PR	HER2	Subtype	Tumor	Muc1
T47D	+	+	-	Luminal A	IDC	+
ZR-75-1	+	+/-	-	Luminal A	IDC	+
MCF-7	+	+	-	Luminal A	IDC	+
BT-20	-	-	-	Triple-negative A	IDC	+
MDAMB231	-	-	-	Triple-negative B	AC	-

Table S2: Characterization and classification of used cell lines based on the status of three important receptors conventionally used in breast cancer subtyping: estrogen receptor (ER), progesterone receptor (PR), and human epithelial receptor 2 (HER2). Luminal A, luminal B, HER2 positive, and triple-negative subtypes, with triple-negative cells are further divided into A and B to capture the heterogeneity and provide an easy link to the widely used names, i. e. , basal A and B.

Table S2. Heavy and Light Chain Primers of MUC1-C binding clones.

HEAVY			
SKM1 No.	PRIMER		ENZYME
	Variable region FWD	Variable region REV	
2	CAGGTGCAGCTGGTAGAGTCT	TGAGGAGACGGTGATCGTGGT	KpnI,NheI
6	CAGGTGCAGCTGGTGCAGTC	TGAGGAGACGGTGACCGTGG	KpnI,NheI
7	CAGATGCAGCTGGTACAGTCTGG	TGAGGAGACGGTGACCGTGG	KpnI,NheI
13	CAGGTGCAGCTGGTGCAGTC	TGAGGAGACGGTGACCGTGG	KpnI,NheI
20	CAGATGCAGCTGGTGCAGTC	TGAGGAGACGGTGACCGTGG	KpnI,NheI
LIGHT			
SKM1 No.	PRIMER		ENZYME
	Variable region FWD	Variable region REV	
2	GATATTGTGATGACCCAGACTCCA	TTTGATATCCAGTCGTGTCCCTTG	BamI,BsiWI
6	AATTTTATGCTGACTCAGCCCGC	TAGGACGGTGACCTTGGTCC	
7	GACATCCAGATGACCAGTCTCT	TTTGATCTCCAGCTTGGTCCCC	
13	GACATCCAGATGACCCAGTCTCC	TTTGATCTCCACCTTGGTCCCT	
20	CAGTCTGCCCTGACTCAGCC	TAGGACGGTCAGCTTGGTCC	

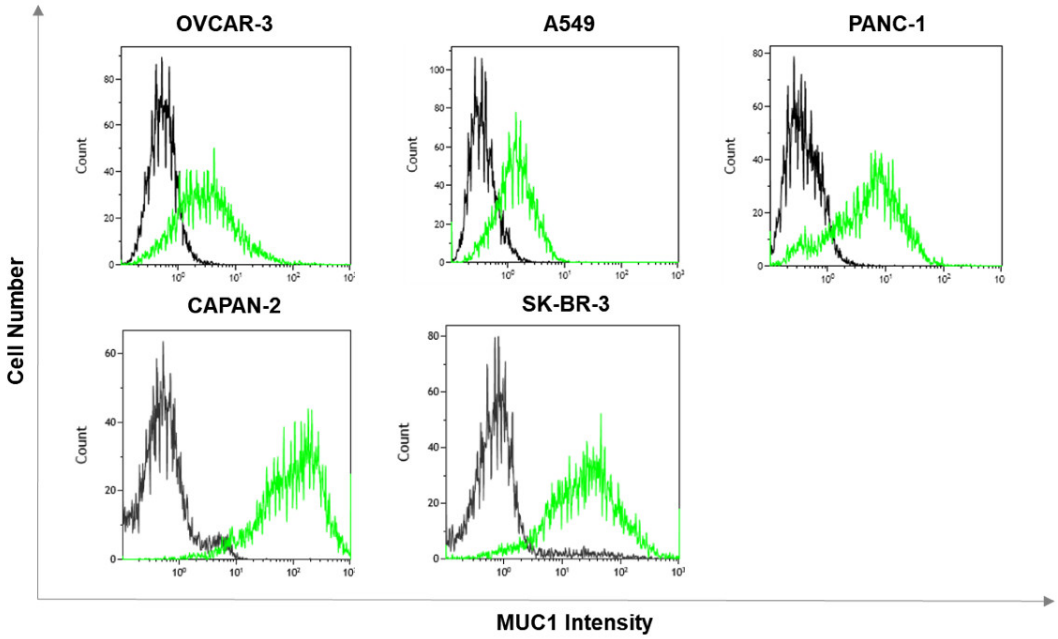

Figure S1. Analysis of MUC1-positive various cancer cell lines.

Muc1 on the surfaces of various cells was measured by flow cytometry with control 2nd antibody (Black line) or Muc1 N-terminal domain(full-length Muc1) antibody(Green line).

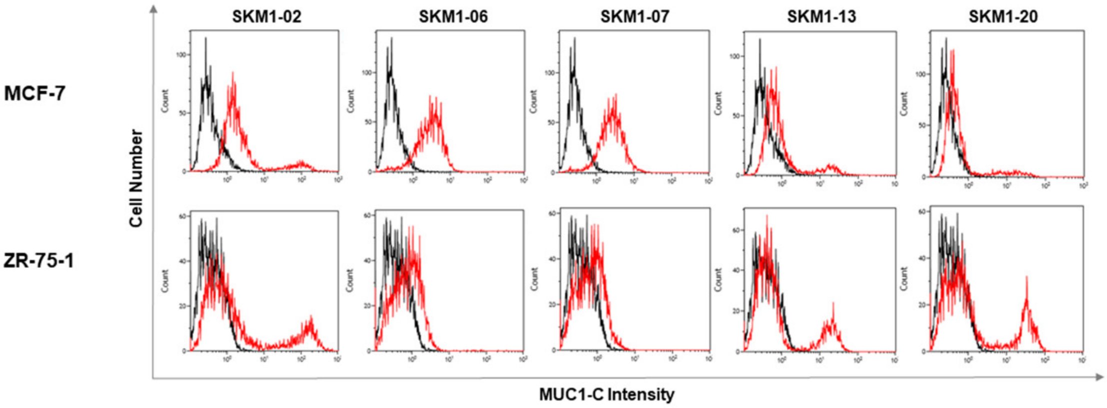

Figure S2. Derivation of ScFv clones through specific binding to Muc1 overexpressing cells.

Specific interaction of Muc1 candidate ScFv with Muc1 on Breast cancer cells. The existence of Muc1 on the surfaces of Breast cancer was measured by flow cytometry with control antibody (Black line) or indicated Muc1 clone ab (Red Line)
